# Internet and depression in adolescents: Evidence from China

**DOI:** 10.3389/fpsyg.2023.1026920

**Published:** 2023-02-13

**Authors:** Yuna Ma, Jiafeng Gu

**Affiliations:** ^1^School of Social Work, China Youth University for Political Sciences, Beijing, China; ^2^Institute of Social Science Survey, Peking University, Beijing, China

**Keywords:** internet, adolescents, depression, online activity, logistic regression

## Abstract

Despite growing attention to Internet activity as a social determinant of depression in adolescents, few studies have focused on its diverse effects on depressive symptoms. Using data from the 2020 China Family Panel Study, this study employed logistic regression analysis to examine how Internet activity affects depressive symptoms in adolescents in China. The results indicated that adolescents with longer online duration using mobile phones tended to have higher levels of depression. Adolescents who engaged in online activities related to games, shopping, and entertainment had more severe depressive symptoms, but their time spent on online learning was not significantly associated with their level of depression. These findings suggest a dynamic link between Internet activity and adolescent depression and offer policy implications for addressing depressive symptoms in adolescents. Specifically, Internet and youth development policies and public health programs during the COVID-19 pandemic should be designed based on a comprehensive account of all aspects of Internet activity.

## 1. Introduction

Internet activity and depression are topics of interest to a broad range of health researchers and policymakers and the general public. An increasing number of studies have shown that Internet activity is a fundamental social determinant of depression (Sayeed et al., 2020) with direct and indirect effects on depression in adolescents (Karamitsa and Skordilis, 2015). Excessive involvement in Internet activity can destroy someone’s life and mental health through mental harassment, cyber intimidation, privacy infringement, which often result in decreased social participation (Singh, 2020). These abhorrent impacts are of particular concern when it comes to adolescents due to their fragile developmental state. Further complicating matters, during the COVID-19 epidemic, online classes and the use of electronic devices greatly increased among adolescents, which adversely affects their depressive symptoms (Marci et al., 2022). That said, owing to the complexity of Internet activity, its precise impact mechanism on depression has not yet been clarified and a general consensus has yet to be reached. Thus, the relationship between Internet activity and depression in adolescents remains unclear. An individual’s level of depression, which reflects his/her mental health status, is considered a sensitive and holistic measure of overall depression (Bain et al., 2020). However, to date, although it is known that an individual’s level of depression depends on the influence of Internet activity, their relationship has not been completely scientifically explained.

This study aimed to determine the connections between adolescents’ levels of depression and their online activities. Cognition about the connections between certain Internet activities and individuals’ levels of depression can be crucial for developing Internet and public health programs that promote adolescent health after COVID-19. As the largest developing country, China has the biggest population of adolescents in the world and is actively implementing measures for the reform and governance of Internet use to improve Internet activity as part of the implementation of a healthy China strategy. Thus, China represents is a key area for research on Internet activity and depression among adolescents. This study used data from the 2020 China Family Panel Study to examine the impact of Internet activity on adolescents’ levels of depression and to determine the impact mechanism.

This paper is organized as follows: Section 2 presents the literature review and hypothesis development; Section 3 presents the data, samples, and methods; Section 4 presents the results; Section 5 presents the discussion; and Section concludes the study.

## 2. Literature review and hypothesis development

In 1998, Kraut et al. published one of the first studies on the issue of the Internet and mental health, the results of which indicated that online activities are linked to a reduction in communication between family members and an increase in depressive symptoms (Kraut et al., 1998; Banjanin et al., 2015). Problematic Internet use is characterized by poorly controlled preoccupations, urges, or behaviors regarding computer usage and Internet access that lead to impairment or distress (Aviv et al., 2010). Therefore, governments in various countries have been actively seeking to reduce Internet use to reduce depressive symptoms and other mental health problems in adolescents (Chung et al., 2019). Mental health professionals are encouraged to promptly evaluate and develop preventive interventions for depressive symptoms in adolescents by monitoring and managing their online time (Wu et al., 2022). However, a large gap still exists in our knowledge of the links and pathways between Internet activity and adolescent depression (Lau et al., 2018). Research on Internet activity and depression in Western countries commenced earlier than other regions and has been relatively comprehensive thus far, having mainly investigated the impacts of different Internet activities on depression and the time spent online (Rabadi et al., 2017). However, there is a lack of such research in China, the world’s second-largest economy, which is not commensurate with the current world pattern. According to a study involving a sample of 6,468 10–18-year-old adolescents recruited from local schools in 2015 in Guangzhou, China, the five highest-ranked online activities were social networking (94.73%), school work (86.53%), entertainment (82.44%), Internet gaming (73.42%) and online shopping (33.67%) (Xin et al., 2018). As the largest developing country in the world, China’s experience with Internet use, adolescent development, and health improvement has important implications and value for a vast number of developing countries. Therefore, the empirical testing of relevant hypotheses concerning the relationship between Internet activity and depression in Chinese adolescents has important theoretical and practical significance.

Adolescents spend a significant amount of time using the Internet for recreational and academic purposes (Khan et al., 2021). While the Internet can increase social interaction and peer-to-peer support, excessive use has been linked to psychosocial problems, such as depression (Khan et al., 2021). A study of high school students in Belgrade, Serbia, revealed a statistically significant positive correlation between the level of Internet addiction and depressive symptoms (Banjanin et al., 2015). In another study, spending too much time on the Internet was related to a decline in social interaction, feelings of sadness, loss of interest, and poor sleep quality (Ko et al., 2005). In a study by Rabadi et al. (2017), the amount of time spent online revealed distinctive usage patterns—the Internet addicted group reported a higher rate of participation in online shopping, community activities, and games, whereas non-addicted participants reported a higher rate of conducting information searches, using e-mail, and chatting.

Over the past two decades, with the development of information technology, many new issues have emerged regarding the potential connection between Internet use and certain mental health problems such as depression (Pantic, 2014). By 2021, the number of Internet users among adolescents had reached 256 million, accounting for 45% of all Internet users in China (CINIC, 2021c). Furthermore, the average time spent on various online activities has recently increased, with 20.7 h online per week in 2021 T (CINIC, 2021c). The main information access channels for adolescents in 2021 in China were mobile phones, desktop computers, and laptops: 86.3, 71.2, and 51.2%, respectively (CINIC, 2021c). Some research has shown that adolescents who spend more time online have a higher risk of experiencing depression symptoms in China (Wu et al., 2022). Problematic smartphone use is also a risk factor for adolescent depression (Wang J. L. et al., 2019). However, there is currently no consensus on the influence of Internet usage with computers on depression. In our study, we tested the relationship between the time spent online with different information access methods and depressive symptoms in adolescents. Thus, based on the previously published data, the following two hypotheses are proposed:

*Hypothesis 1*: Online duration using mobile devices is positively correlated with the probability of getting depressed in adolescents.

*Hypothesis 2*: Online duration using computers is positively correlated with the probability of getting depressed in adolescents.

Online games are related to adolescent depression levels(Hellström et al., 2015). More intensive and frequent online gaming experiences are significantly and negatively related to gamers’ mental health (Chan and Cheung, 2020), and a higher online game addiction tendency is often accompanied by emotional problems, such as depressive disorder (Hong et al., 2018). Prior research has demonstrated a significant relationship between the intensity of playing video games with the depression levels of teenagers aged 15–18 years: those who had played video games for more than 2 h a day had a higher mean Children’s Depression Inventory-2 (CDI-2) score when compared to those who played video games for less than 2 h per day (Humries et al., 2020). In 2021, China had 509 million online gamers, which is the largest game population in the world (CINIC, 2021b). Online gaming has become a popular method for adolescents in China to spend their free hours (Kramer and Landsberger, 2015). One study showed that online games accounted for 73.42% of all online activities among adolescents aged 10–18 years in Guangzhou, China (Xin et al., 2018), while another found that mobile game addiction is positively associated with depression in Chinese adolescents (Wang J. L. et al., 2019). Thus, based on the previously published data, the following two hypotheses are proposed:

*Hypothesis 3*: Adolescents who play online games are more likely to have depression than those who do not play online games.

*Hypothesis 4*: Adolescents who play online games almost everyday are more likely to have depression than those who play online games less than everyday.

Online shopping is relevant to Internet addiction in adolescents and is positively related to the level of depression (Seki et al., 2019). Research has found that online shopping predicts depression in adults and vice versa (Keskin and Günü, 2017). However, little attention has been paid to the relationship between online shopping and depression in adolescents. By December 2021, the number of online shopping users in China had reached 842 million, up from 59.68 million in December 2020, accounting for a staggering 81.6 percent of all Internet users worldwide 91.6(CBIRI, 2021). In one survey, online shopping was found to account for 33.67% of all online activities among adolescents aged 10–18 years in Guangzhou, China (Xin et al., 2018). Researchers in China generally agree that Internet pushing shopping will reduce subjective happiness and may lead to depression among college students in China (Yu et al., 2019). Wang et al. (2022) found that online shopping addiction was significantly and positively associated with negative emotions among adolescents during the COVID-19 pandemic in China (He et al., 2019). Thus, based on the previously published data, the following two hypotheses are proposed:

*Hypothesis 5*: Adolescents who shop online are more likely to have depression than those who do not shop online.

*Hypothesis 6*: Adolescents who do online shopping almost everyday are more likely to have depression than those who do online shopping less than everyday.

Adolescents increasingly use the Internet for entertainment to varying degrees, which may make them prone to Internet addiction (Karacic and Oreskovic, 2017). A simple random sample of 1,078 adolescents aged 11–18 years attending elementary and secondary schools in Croatia, Finland, and Poland showed that adolescents mostly use the Internet for entertainment (84%) (Karacic and Oreskovic, 2017). However, there is evidence that online entertainment is closely related to depressive symptoms; for example, the frequency of social networking site usage for entertainment was successfully associated with depression and psychological distress symptoms in undergraduate students in Jordan after controlling for demographics (Al-Dwaikat et al., 2020). In contrast, little attention has been paid to the relationship between online entertainment and depression among adolescents. As aforementioned, online entertainment accounts for 82.44% of all online activities in adolescents aged 10–18 years in Guangzhou, China (Xin et al., 2018). During the COVID-19 pandemic, 33.5% of Chinese adolescents reported spending more time on online entertainment. As an emerging form of online entertainment, the proportion of adolescents watching short video clips in China has increased from 40.5% in 2018 to 49.3% in 2020 (CINIC, 2021a). Thus, based on the previously published data, the following two hypotheses are proposed:

*Hypothesis 7*: Adolescents who watch short videos or live online platform programs are more likely to have depression than those who do not watch short videos or live online platform programs.

*Hypothesis 8*: Adolescents who watch short videos or live online platform programs almost everyday are more likely to have depression than those who watch short videos or live online platform programs less than everyday.

The COVID-19 pandemic poses a serious challenge to adolescents’ daily lives, including schooling and learning, and greatly impacts their mental health (Karacic and Oreskovic, 2017). In contrast to previous Internet usage patterns, participants who demonstrated a higher rate of online learning, such as online information searches, were classified as non-addicted Internet users (Rabadi et al., 2017). A prior study of undergraduate students in Jordan during the COVID-19 lockdown found that academic use of social networking sites is significantly negatively correlated with depression (Al-Dwaikat et al., 2020), indicating that online learning plays a role in partially relieving depression. Other research has found that learning through online platforms has given rise to depression disorders among undergraduate university students (Fawaz and Samaha, 2021).

According to the CAC (2021), the Internet access rate of primary and secondary schools in China (including teaching sites) rose from 79.37% in 2016 to 100% in 2020 (CINIC, 2021a). In particular, the COVID-19 outbreak in early 2020 delayed the opening of many schools across the country. To ensure that classes were not suspended, many schools shifted their courses from offline to online formats, with 89.9% of underage Internet users regularly using the Internet to study (CINIC, 2021a). In China, the association between academic stress in online learning and depression was found to be stronger among females compared with males among adolescents during the COVID-19 pandemic (She et al., 2021). Thus, based on the previously published data, the following two hypotheses are proposed:

*Hypothesis 9*: Adolescents who use online learning are less likely to have depression than those who do not.

*Hypothesis 10*: Adolescents who do online learning almost everyday are less likely to have depression than those who do online learning less than everyday.

## 3. Methods

### 3.1. Sample

The data for this study were obtained from the China Family Panel Studies (CFPS), a comprehensive survey based on individuals, families, and communities conducted by the China Social Science Research Center (ISSS) at Peking University. CFPS data are widely used in research in China’s health fields. The CFPS sample comprised 25 provinces, municipalities, and autonomous regions in mainland China, excluding Xinjiang, Tibet, Qinghai, Inner Mongolia, Ningxia, and Hainan, and the sample population accounted for 95% of mainland China’s population. The CFPS employs a multi-stage probability sample drawn using implicit stratification, and the CFPS sample can be regarded as a nationally representative sample. Specifically, the samples of each sub-sample box are extracted through three stages. The first-stage sample (PSU) is an administrative district/county, the second-stage sample (SSU) is an administrative village/neighborhood committee, and the third-stage (terminal) sample (TSU) is a family household. The sampling in the first two stages of CFPS uses official administrative division data. In the third stage, the map address method is used to construct the end sampling frame, and the sample households are selected by the cyclic equidistant sampling method with random starting points. During the special historical period of coronavirus disease 2019 (hereafter, COVID-19), the 2020 CFPS survey was conducted using computer-assisted telephone interviews or online interviews *via* computer-assisted online interviews. This study also used personal questionnaires from a 2020 follow-up study. The dataset included 3,101 adolescents aged 9–18 years, for a total of 28,590 people. Adolescent samples with missing values were removed. The exclusion criteria included those with missing sociodemographic information, those with missing items in the depression scale, and those unable to obtain depressive symptoms data. The final adolescent sample (*n* = 2,158) provided the data for this study.

### 3.2. Measures

#### 3.2.1. Dependent variables of depression

The Center for Epidemiologic Studies Depression Scale (CES-D) was used in 2020 CFPS to measure an individual’s level of depression. Since 2016, the simplified mode of the CES-D scale (the CESD8) has been used, with the number of questions reduced from 20 to 8. The CESD8 is widely employed to screen depressive symptoms with good reliability and validity and has been proven to be suitable for Chinese residents. Higher scores indicate more severe depressive symptoms. According to relevant research, a score above 14 points indicates that the respondent has depressive symptoms (Gu, 2022). The operational definition of the depression variable was as follows: a score above 14 points was defined as 1 and otherwise 0.

#### 3.2.2. Internet activity

Regarding the time spent online, respondents were asked to indicate (in minutes) the following two questions regarding their Internet use: “In general, how much time do you use your mobile device to surf the web every day?” and “In general, how long do you use your computer to surf the web every day?” Therefore, these two variables are continuous variables. Regarding online games, respondents were asked, “Have you played online games in the past week?” Those who responded “No” were assigned to the baseline group. Regarding the frequency of playing online games, respondents were asked, “How often did you play online games in the past week?” Those who answered “Less than everyday” were assigned to the baseline group. Regarding online shopping, respondents were asked, “Have you shopped online games in the past week?” Those who indicated

No” were assigned to the baseline group. Regarding the frequency of online shopping, respondents were asked, “In the past week, have you shopped online almost every day?” Those who answered “Less than everyday” were assigned to the baseline group. Regarding online entertainment, respondents were asked, “In the past week, have you watched short videos or live online platform programs?” Those who responded “No” were assigned to the baseline group. Regarding the frequency of online shopping, respondents were asked, “In the past week, how often did you watch short videos or live online platform programs almost every day?” Those who indicated “Less than everyday” were assigned to the baseline group. Regarding online learning, respondents were asked, “In the past week, have you used e-learning?” Those who answered “No” were assigned to the baseline group. Regarding the frequency of online learning, respondents were asked, “In the past week, have you used e-learning almost every day?” Those who responded “Less than everyday” were assigned to the baseline group. Therefore, these variables were categorical variables.

#### 3.2.3. Control variables

The control variables were information access channels, relationships, happiness, gender, age, health status, changes in health, study-induced stress, romantic relationships, and education level. Regarding information access channels, respondents were asked to indicate on a 5-point Likert scale the answers to following questions: “How important is mobile text messaging to access information?” and “How important is the Internet to access information?” A response of “1” means very unimportant, and “5” means very important. Regarding relationships, respondents were asked to indicate on a 10-point Likert scale the question, “How good do you think relationships are?” A response of “0” means poor, and an answer of “10” means excellent. Similarly, regarding happiness, a 10-point scale was used to answer the question, “How happy do you think you feel?,” where “0” indicates extreme unhappiness and “10” means extreme happiness. Regarding gender, females were assigned to the baseline group. Regarding personal health status, respondents were asked, “How healthy do you think you are?” The possible choices were extremely healthy, very healthy, relatively healthy, average, and unhealthy. The average and unhealthy values were set as the baseline values. Regarding changes in personal health, respondents were asked, “How do you think your health is compared to a year ago?,” with three options: were got better, no change, and got worse. Those who reported “No changes” were assigned to the baseline group. Regarding study-induced stress, respondents were asked, “How much pressure do you think you have from studying?,” measured on a 5-point Likert scale where “1” = no pressure and “5” = a lot of pressure. Regarding romantic relationships, respondents were asked, “Have you ever been in love?” Those who answered “Yes” were assigned to the baseline group. Finally, respondents reported their education levels as (0) primary school, (1) junior high school, (2) high school, or (3) college. Those with primary school education were assigned to the baseline group.

### 3.3. Statistical modeling

The binary correlation between categorical explanatory variables and depression in adolescents was described using chi-squared analysis. The data were cleared and processed using StataMP 16. A multivariate logistic regression was used to test each hypothesis in the theoretical model.

As the dependent variable in this study was a binary variable, binary logistic regression was used as the statistical model:


$$\text{logit}\frac{p}{1-p}=\alpha +B_{1}x_{1}+B_{2}x_{2}+\cdots \cdots +B_{i}x_{i}$$

where p was the probability of depression, $\frac{p}{1-p}$ was the occurrence ratio or probability of depression relative to no depression, $x_{1},x_{2,}\ldots ,x_{i}$ were the core explanatory variables and related control variables, $B_{i}$ was the regression coefficient, and α was the intercept term.

## 4. Results

The adolescent sample characteristics are listed in Table 1, which shows four aspects of Internet activity (games, shopping, entertainment, and learning) and their correlations with depression. In 2020, 23.77% (*n* = 513) and 76.23% (*n* = 1,645) of respondents had and did not have depressive symptoms, respectively.

**Table 1:** Distribution of categorical variables.

	Number of Participants (*n* = 2,158)		Depression		*x*^2^(*p*)
	Classification	*N* (%)	No *N* (%)	Yes *N* (%)	
Online games	No	791 (36.65)	625 (37.99)	166 (32.36)	5.348^**^ (0.021)
	Yes	1,367 (63.35)	1,020 (61.01)	347 (67.64)	
Frequency of online games	Less than everyday	1,698 (78.68)	1,296 (78.78)	402 (78.36)	0.041 (0.839)
	Almost everyday	460 (21.32)	349 (21.22)	111 (21.64)	
Online shopping	No	1,447 (67.05)	1,134 (68.94)	313 (61.01)	11.11^***^ (0.001)
	Yes	711 (32.95)	511 (31.06)	200 (38.99)	
Frequency of online shopping	Less than everyday	2,142 (99.26)	1,632 (99.21)	510 (99.42)	0.224 (0.636)
	Almost everyday	16 (0.74)	13 (0.79)	3 (0.58)	
Online entertainment	No	397 (18.4)	314 (19.09)	83 (16.18)	2.204 (0.138)
	Yes	1,761 (81.6)	1,331 (80.91)	430 (83.82)	
Frequency of online entertainment	Less than everyday	1,374 (63.67)	1,083 (65.84)	291 (56.73)	14.033^***^ (0.001)
	Almost everyday	784 (36.33)	562 (34.16)	222 (43.27)	
Online learning	No	1,200 (55.61)	930 (56.53)	270 (52.63)	2.414 (0.12)
	Yes	958 (44.39)	715 (43.47)	243 (47.37)	
Frequency of online learning	Less than everyday	1,831 (84.85)	1,401 (85.17)	430 (83.82)	0.552 (0.458)
	Almost everyday	327 (15.15)	244 (14.83)	83 (16.18)	
Gender	Female	1,005 (46.57)	746 (45.35)	259 (50.49)	4.149^**^ (0.042)
	Male	1,153 (53.43)	899 (54.65)	254 (49.51)	
Health status	Average or unhealthy	53 (2.46)	28 (1.7)	25 (2.46)	45.623^***^ (0.001)
	Relatively healthy	649 (30.07)	453 (27.54)	196 (38.21)	
	Very healthy	690 (31.97)	536 (32.58)	154 (30.02)	
	Extremely healthy	766 (35.5)	628 (38.18)	138 (26.9)	
Health changes	No change	1,191 (55.19)	885 (53.8)	306 (59.65)	36.261^***^ (0.001)
	Got better	823 (38.14)	673 (40.91)	150 (29.24)	
	Got worse	144 (6.67)	87 (5.29)	57 (11.11)	
Romantic relationship	No	2,057 (95.32)	1,583 (96.23)	474 (92.4)	12.881^***^ (0.001)
	Yes	101 (4.68)	62 (3.77)	39 (7.6)	
Education	Primary school	523 (24.24)	413 (25.11)	110 (21.44)	2.592 (0.459)
	Junior high school	833 (38.6)	632 (38.42)	201 (39.18)	
	High School	678 (31.42)	504 (30.64)	174 (33.92)	
	College	124 (5.75)	96 (5.84)	28 (5.46)	
Depression	No	1,645 (76.23)			
	Yes	513 (23.77)			

***p < 0.01; **p < 0.05; *p < 0.1.

Table 2 shows the multivariate logistic regression analysis results with time spent online, four types of Internet activity, and the control variables. People who spent more time online on mobile devices every day had higher levels of depression than those who spent less time online (OR:1.001; 95% CI:0.999–1.001). Thus, Hypothesis 1 was confirmed. Next, we examined the relationship between Internet activity and depression in adolescents from the integrated perspective of Internet activity. The sum of separate Internet activities provides a systematic explanation for the influence of online personal activities on adolescent depression. Those who had played games online in the past week reporting having more depressive symptoms than those had not (OR:1.337; 95% CI:1.033–1.731). Thus, Hypothesis 3 was confirmed. In addition, those who had shopped online in the past week tended to be more depressed than those who had not shopped online in the past week (OR:1.247; 95% CI:0.977–1.592). Thus, Hypothesis 5 was confirmed. Further, those who had watched short videos or used live online platform programs almost every day in the past week reported more depressive symptoms than those who had not (OR:1.337; 95% CI:1.04–1.719). Thus, Hypothesis 8 was confirmed.

**Table 2:** Multivariable logistic regression analysis.

Characteristics	Odds ratio	Drink_Y_18 95% CI	*p*-value
Online duration *via* mobile	1.001^*^	0.999–1.001	0.09
Online duration *via* computer	0.998	0.997–1.001	0.191
Online game (Re: No)			
Yes	1.337^**^	1.033–1.731	0.028
Frequency of online games (Re: less than everyday)			
Almost everyday	0.837	0.662–1.125	0.238
Online Shopping (Re: No)			
Yes	1.247^*^	0.977–1.592	0.076
Frequency of online shopping (Re: less than everyday)			
Almost everyday	0.354	0.089–1.415	0.143
Online entertainment (Re: No)			
Yes	0.884	0.648–1.206	0.436
Frequency of online entertainment (Re: less than everyday)			
Almost everyday	1.337^**^	1.04–1.719	0.023
Online learning (Re: No)			
Yes	1.137	0.891–1.45	0.302
Frequency of online learning (Re: less than everyday)			
Almost everyday	1.125	0.806–1.57	0.49
Mobile text messaging for information	1.148^***^	1.045–1.261	0.004
Internet for information	1.061	0.949–1.185	0.301
Relationship	0.895^***^	0.841–0.952	0.001
Happiness	0.82^***^	0.773–0.871	0.001
Gender (Re: female)			
Male	0.839	0.659–1.067	0.152
Age	1.073	0.963–1.196	0.201
Health status (Re: average or unhealthy)			
Relatively healthy	0.639	0.343–1.192	0.159
Very healthy	0.514^**^	0.273–0.968	0.039
Great health	0.474^**^	0.25–0.899	0.022
Changes in health (Re: no change)			
Got better	0.687^***^	0.541–0.872	0.002
Got worse	1.645^**^	1.109–2.439	0.013
Study-induced stress	1.444^***^	1.296–1.608	0.001
Romantic relationship (Re: Yes)			
No	0.477^**^	0.298–0.763	0.02
Education (Re: illiterate/semi-literate)			
Primary school	0.785	0.521–1.181	0.246
Junior high school	0.528^*^	0.275–1.014	0.055
High School	0.34^**^	0.139–0.829	0.018
Constant	0.936	0.189–4.621	0.935
LR chi-squared	519.298(0.000)		
-2Log likelihood	2107.408		
Cox and Snell R square	0.113		
MacFadden square	0.11		
Nagelkerke square	0.19		

***p < 0.01; **p < 0.05; *p < 0.1.

In 2020, respondents who thought mobile text messaging was more important to access information had more depressive symptoms (OR:1.148; 95% CI:1.045–1.261). People who had many of relationships were less prone to depression than those with few relationships (OR:0.895; 95% CI:0.841–0.952). People who felt happier were less likely to be depressed (OR:0.82; 95% CI:0.773–0.871). Respondents who felt they were extremely healthy were less likely to be depressed than those who felt they had average health or were unhealthy (OR:0.514; 95% CI: 0.273–0.968), and those who felt they were in great health were less prone to depression than those who felt they were unhealthy or had average health (OR:0.474; 95% CI 0.25–0.899). Individuals whose health had improved compared to a year ago were not likely to be depressed compared to those who thought their health status had not changed (OR:0.687; 95% CI:0.541–0.872). Individuals whose health had deteriorated compared to a year ago were more likely to be depressed than those who thought their health had not changed (OR:1.645; 95% CI:1.109–2.439). Respondents with more stress due to studying tended to be depressed than those with less stress from studying (OR,1.444; 95% CI,1.296–1.608). Those not in a romantic relationship had lower levels of depression than those in one (OR:0.477; 95% CI:0.298–0.763). Those in junior high school had lower depression levels than those in primary school (OR,0.528; 95% CI,0.275–1.014; OR, 0.785; 95% CI, 0.521–1.181), and those in high school had lower levels of depression than those in primary school (OR:0.34; 95% CI: 0.139–0.829; OR:0.785; 95% CI: 0.521–1.181).

## 5. Discussion

Some studies have examined the relationship between Internet activity and depression in adolescents in Western countries. However, equivalent research in China is lacking. The results of this study show that adolescents with higher mobile device use have higher levels of depressive symptoms. Thus, Hypothesis 1 was confirmed. This conclusion is consistent with those of related studies conducted in Western countries (Wang P. et al., 2019). The positive impact of time spent online on mobile devices on depression in adolescents is universal. However, no relationship exists between online duration using computers and depression. Thus, Hypothesis 2 was not confirmed. Internet searches with smartphones now exceed desktop searches in adolescents using search engines in China (CINIC, 2015). Compared with traditional computer platforms, smartphone platforms not only provide the basic functions of traditional computer platforms but also have incomparable advantages, such as mobility, portability, and ease of Internet use (Ha et al., 2007). Simultaneously, Internet usage with smartphones can be a clever way to fill users’ time gaps (Li and Counts, 2007). These advantages have led to a large number of users switching from traditional computer platforms to emerging smartphone platforms (Ha et al., 2007). However, with the popularity of smartphone platforms, inappropriate use behaviors (such as Internet addiction) have greater psychological consequences than Internet usage with computers. For example, smartphone games, which are closely related to depression, are far more addictive than computer games. The positive relationship between time spent online using mobile devices and depression shows that the aggregate Internet usage patterns of mobile devices—gaming, shopping, entertainment, and learning online—provide the social conditions for the worsening depression in adolescents during the COVID-19 pandemic.

Playing online games is closely related to mental health (Humries et al., 2020). The negative impact of online games has received considerable attention and has become a popular research topic in recent years (Wan and Chiou, 2006). The results of this study verify a significant relationship between online game playing and depressive symptoms in adolescents, which is in line with previous studies. Thus, Hypothesis 3 was confirmed. Research subjects who play online games had higher levels of depression than those who did not play online games. This confirms that playing online games contributed significantly to depressive symptoms among adolescents during the COVID-19 pandemic. However, there was no significant relationship between the frequency of playing online games and depressive symptoms (Chan and Cheung, 2020). Thus, Hypothesis 4 was not confirmed. This might be because the intensity of some (positive) components in online gaming buffers against the negative effect of the intensity of some other components in online games on gamers’ mental health. Some components of online gaming negatively impact mental health for people who engage in high frequency, high intensity online gaming (Chan and Cheung, 2020). Nevertheless, it should be acknowledged that online games, as a means of stimulating excitement, fun, and socialization, can be a valuable tool for preventing emotional disorders in adolescents (David et al., 2021).

Online shopping is closely related to depression in adults (Keskin and Günü, 2017). Similarly, compulsive buying entails all the elements of addictive behavior in youth and can lead to psychological problems such as guilt, anxiety, and depression (Cassidy and Adair, 2021). Interest in this area has been reignited by the growth in online shopping during the recent COVID-19 pandemic (Cassidy and Adair, 2021). However, the impact of online shopping on depression among adolescents has not received sufficient attention. The results of this study confirm a significant relationship between online shopping and depressive symptoms in adolescents. Thus, Hypothesis 5 was confirmed. Research subjects who shop online have higher levels of depression compared to those who do not. This confirms that adolescent online shopping was a significant factor in the development of depressive symptoms during the pandemic. However, there no significant relationship could be identified between the frequency of online shopping and depressive symptoms. Thus, Hypothesis 6 was not confirmed. This particular result appears to be the reverse of what might be expected from Western research; that is, it might be reasonable to predict that people with a severe degree of online shopping addiction have higher depression scores compared to people with moderate or inexistent addiction (Mitrofan, 2015) because those who are likely to shop online lack social interaction in traditional markets. This might be because the intensity of different types of interactions in online shopping can buffer against the negative effect of the intensity of some components in online shopping on gamers’ mental health. With the increased prevalence of live streaming services that provide a new shopping experience, consumers have been strongly encouraged to engage in impulse online buying during the recent COVID-19 pandemic in China. It may be that those who are prone to shop on live streaming platforms have more viewer–broadcaster interactions and viewer–viewer interactions, which positively affects viewers’ sense of being part of a virtual community (He et al., 2022).

Online entertainment is closely related to Internet addiction and mental health in adolescents (Bezinovic et al., 2015). The results of this study show that there is no significant relationship between online entertainment and depressive symptoms among adolescents. Thus, Hypothesis 7 was not confirmed. However, a significant relationship was found between the frequency of online entertainment and depressive symptoms. Thus, Hypothesis 8 was confirmed. When people spend more time watching short videos or using live online platform programs, negative effects on depressive symptoms in adolescents are likely to occur. This confirms that the intensity of online entertainment among adolescents contributed significantly to depressive symptoms during the pandemic, which is consistent with the findings of previous studies (Hoare et al., 2017). Limited access to short videos or live online platform programs is currently a major challenge for adolescents under the influence of the COVID-19 pandemic, which has increased online entertainment. With the recent development of artificial intelligence technology and the arrival of the 5G era, the short video industry has boomed in China (Liu, 2022). A study on the impact of the COVID-19 pandemic on China’s entertainment industry showed that the online entertainment market, including streaming platforms, boomed as people were confined to their homes (González-González et al., 2021). Our research further proves the need for rational usage of online entertainment during the COVID-19 pandemic in adolescents to preserve their mental health.

Online learning has been adopted internationally as an alternative teaching or learning strategy during COVID-19 quarantine measures to fill the academic gap created by the existing reality of the pandemic owing to nationwide school closures (Fawaz and Samaha, 2021). The results of this study show that there is no significant relationship between online learning and depressive symptoms among adolescents. Thus, Hypothesis 9 was not confirmed. Similarly, the study results indicated that there was no significant relationship between the frequency of online learning and depressive symptoms in adolescents. Thus, Hypothesis 10 was not confirmed. Previous research found a significant relationship between perceived difficulties in online learning and depression among senior high school students in Jordan during the COVID-19 pandemic. However, the present study confirms that during the COVID-19 pandemic, the time spent on online learning neither significantly caused nor alleviated depressive symptoms in adolescents in China. This might be because the online interaction between teachers and students and among students alleviates the stressful load inherent in online learning, which helps to cushion against the negative effect of perceived difficulties in online learning on students’ mental health.

In addition, this research showed that adolescents who thought mobile text messaging was more important to their information access had more depressive symptoms than those who believed it was unimportant. Moreover, this study demonstrated that the adolescents with better relationships were less prone to depression than those with poor relationships and that those who felt happier were less depressed. Respondents who stated who they were extremely healthy were less likely to be depressed than those who felt they were unhealthy or had average health, while those who reported being in great health were less prone to depression than those felt they had average health or and were unhealthy. Those whose health had improved compared to a year ago were less likely to be depressive than those who thought their health had not changed in the same period, and those who through their health had worsened compared to a year ago were more likely to be depressed than those who thought their health had not change. Respondents with more stress from studying tended to be more depressed than those with less stress. Those not in romantic relationships had lower levels of depression than those in romantic relationships. Finally, those in junior high school had lower levels of depression than those in primary school, and those in high school had lower level of depression than those in primary school.

This study contributes to the literature in three main ways: First, it extends the understanding of the association between Internet activity and depression in adolescents. Second, it expands the social determinants of public health theory by drawing insights obtained from an Internet-based living lens. Third, different dimensions of Internet activity were found to influence depression in adolescents. Some findings do not coincide with the majority of the literature on this topic. Namely, the existence of a counterexample shows that the relationship between Internet activity and depression in adolescents is complex and nonlinear. Therefore, this study helps eliminate people’s mindsets and aids in objectively and comprehensively evaluating the impact of Internet usage on depression. These findings provide important practical guidance for formulating relevant policies.

An important correlation between Internet activity and depression was found among adolescents. However, owing to the questionnaire contents and survey scope, this study has the following limitations. First, due to data limitations, the Internet activity indicators investigated in this research were limited and other factors may have been omitted. Second, because of the attributes of cross-sectional data, it is difficult to determine the precise causal mechanism of Internet activity that leads to depression. Third, the meaning of Internet activities is becoming increasingly complex, and there are often interactions with unique personal characteristics. Thus, the relationship between Internet activity and individual aspects of depression requires further investigation. This study shows that gender and age affect Internet activity, but the interaction variables of these factors are not introduced into the model. Therefore, it was impossible to examine the interaction effects of these factors. This is a research avenue worthy of further study.

## 6. Conclusion

This study used the 2020 CFPS data to empirically study the impact of Internet activity on depression in adolescents. The study found that online duration *via* mobile devices and the following Internet activity affected depression in adolescents. Specifically, online games, online shopping, and the frequency of online entertainment were all associated with higher levels of depression. In addition, mobile text messaging to access information; relationship, happiness, and health status; changes in health; study-induced stress; and education all significantly impacted depression in adolescents. These results have implications for policies seeking to improve Internet regulations, public health programs, and youth development policy improvement after the COVID-19 pandemic to establish healthier Internet behaviors in society and enhance the public health of adolescents in China and other countries. They also inform on the types of interventions required to address problems in Internet usage in the field. Strategies that aim to control problematic Internet usage may be effective and focus on a comprehensive account of different Internet-related aspects. The patterns of Internet activity in China can be classified into more subtypes. We suggest that the Internet activity questions in the CFPS survey should be designed with more detail based on its classification, so that a more in-depth and detailed study can be carried out. Moreover, if the patterns and frequency of Internet activity with different online channels were included in the CFPS survey, we could accurately assess the impact of Internet activity on depression in adolescents. In addition, the effect of predictor variables on the probability of getting depressed in adolescents may also be influenced by some moderating variables. In this case, it is necessary to discuss and test the role of interactions of predictor variables. These are the directions for future research.

## Data availability statement

The datasets presented in this study can be found in online repositories. The names of the repository/repositories and accession number(s) can be found at: https://opendata.pku.edu.cn/dataset.xhtml?persistentId=doi:10.18170/DVN/45LCSO.

## Author contributions

YM and JG conceived the research framework, analyzed and interpreted the data, and drafted the manuscript. JG revised the draft, approved the final version, and was accountable for all aspects of the work. All authors contributed to the article and approved the submitted version.

## Funding

This research was funded by the Social Science Foundation of China (17BSH122).

## Conflict of interest

The authors declare that the research was conducted in the absence of any commercial or financial relationships that could be construed as potential conflicts of interest.

## Publisher’s note

All claims expressed in this article are solely those of the authors and do not necessarily represent those of their affiliated organizations, or those of the publisher, the editors and the reviewers. Any product that may be evaluated in this article, or claim that may be made by its manufacturer, is not guaranteed or endorsed by the publisher.
